# Reprogramming bone progenitor identity and potency through control of collagen density and oxygen tension

**DOI:** 10.1016/j.isci.2022.104059

**Published:** 2022-03-11

**Authors:** Rawiya Al Hosni, Laurent Bozec, Scott J. Roberts, Umber Cheema

**Affiliations:** 1 UCL Centre for 3D Models of Health and Disease, Division of Surgery and Interventional Sciences, University College London, 43-45 Foley St, Fitzrovia, W1W 7TY London, UK; 2 Faculty of Dentistry, University of Toronto, 12 Edward Street, Toronto, ON M5G 1G6, Canada; 3 Department of Comparative Biomedical Sciences, The Royal Veterinary College, 4 Royal College St, NW1 0TU London, UK

**Keywords:** Cell biology, Stem cells research, Developmental biology, Biophysics

## Abstract

The biophysical microenvironment of the cell is being increasingly used to control cell signaling and to direct cell function. Herein, engineered 3D tuneable biomimetic scaffolds are used to control the cell microenvironment of Adipose-derived Mesenchymal Stromal Cells (AMSC), which exhibit a collagen density-specific profile for early and late stage bone cell lineage status. Cell potency was enhanced when AMSCs were cultured within low collagen density environments in hypoxic conditions. A transitional culture containing varied collagen densities in hypoxic conditions directed differential cell fate responses. The early skeletal progenitor identity (PDPN^+^CD146^−^CD73^+^CD164^+^) was rescued in the cells which migrated into low collagen density gels, with cells continuously exposed to the high collagen density gels displaying a transitioned bone-cartilage-stromal phenotype (PDPN^+^CD146^+^CD73^−^CD164^-^). This study uncovers the significant contributions of the physical and physiological cell environment and highlights a chemically independent methodology for reprogramming and isolating skeletal progenitor cells from an adipose-derived cell population.

## Highlights

• Fabrication of a 3D transitional culture to control adipose-derived MSC (AMSC) fate
• AMSC potency is enhanced in low collagen density gels under hypoxic conditions
• Early skeletal progenitor identity of AMSCs is enriched in a low collagen density gel
• Bone-cartilage-stromal identity of AMSCs is enriched in a high collagen density gel

## Graphical abstract

**Figure:**
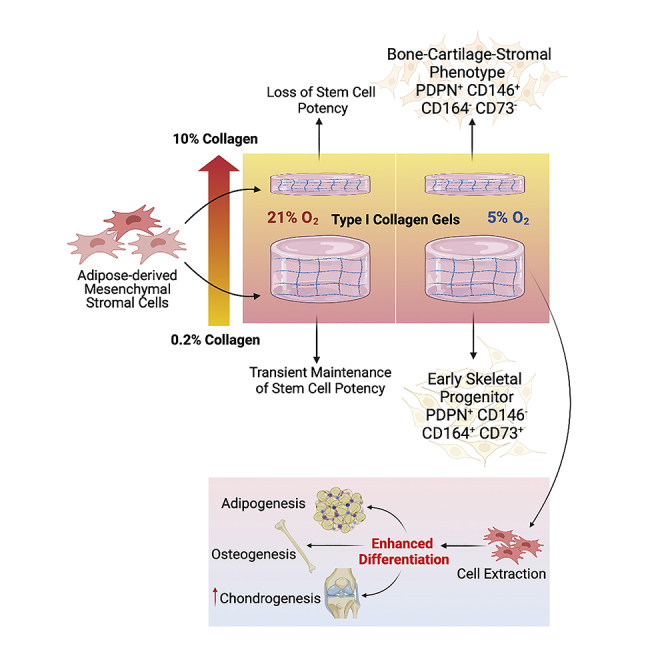

## Introduction

Mesenchymal stromal cells (MSCs) play an important role in physiological and pathological processes. With their inherent plasticity and multilineage potential, understanding the key modulators regulating cell identity and potency is vital. The key features known to regulate cellular behavior include the supporting extracellular matrix (ECM), neighboring cells, soluble signaling factors, physical parameters such as shear stress, stiffness, and topography, and environmental signals such as metabolites and oxygen tension (Mas-Bargues et al., 2019). It is well-known that the modulation of cell characteristics *in vivo* or *in vitro* requires a precise control of both biochemical and biophysical cues (Park et al., 2011; Handorf et al., 2015; Lee et al., 2015). Multiple studies have demonstrated the importance of mechanobiology in directing cellular fate (Guilak et al., 2009; Lee et al., 2015; Robertson et al., 2018; Amer et al., 2020). Indeed, when cells sense a mechanical cue from their environment, this event is converted into a biological response known as mechanotransduction (Handorf et al., 2015; Paluch et al., 2015; Robertson et al., 2018). With the ability to respond to changes in environmental conditions and continuously alter their intrinsic characteristics, it is of importance to study the effect of microenvironmental characteristics on stem/progenitor cell identity and potency.

There is currently a concerted effort to create culture methodologies that better replicate those observed *in vivo*. It is hypothesized that this will better preserve or enhance cell potency *in vitro*. MSCs predominantly reside in collagen type I rich environments, providing structural support and guidance cues that influence cell behavior (proliferation, differentiation, and migration) (Ferreira and Norton, 2012; Cheema and Brown, 2013). Thus, this matrix component may be of importance to mimic the cell-matrix interactions responsible for traction-induced signals to direct the maintenance of cellular behavior (Yamazaki et al., 2010; Abou Neel et al., 2013). In addition, accumulating evidence suggests that MSCs derived from various tissues exhibit tissue-specific properties, posing a critical effect on their differentiation capacity. Indeed, it has been shown that human MSCs maintain a quiescent state when grown on polyacrylamide substrates mimicking the structural and mechanical properties of bone marrow (Winer et al., 2009).

In conjunction with the impact of ECM components in directing cell fate, several studies have reported the influence of oxygen tension on the proliferation and differentiation of stem cells within their native microenvironment (Mohyeldin et al., 2010; Buravkova et al., 2014). It has been hypothesized that low oxygen tensions provide a selective advantage to the maintenance of a continuous population of undifferentiated cells (Mohyeldin et al., 2010). Indeed, bone marrow-derived and adipose-derived MSCs (AMSC) display decreased differentiation capacity when cultured within hypoxic environments while maintaining an undifferentiated population of cells (Khan et al., 2007; Buravkova et al., 2014).

Taken together with the effect of the ECM on cell behavior, these data suggest that cell identity, potency, and fate can be modulated through the control of environmental cues. Furthermore, this presents the intriguing possibility that cells may be reprogrammed through the modification of substrate structural and mechanical properties and surrounding oxygen tension, without the addition of exogenous growth factors or manipulation of gene transcription. Indeed, with relevance to bone biology, it has recently been shown that skeletal stem cells (SSCs) can be isolated from induced tissues through the addition of BMP2 to human AMSCs (Chan et al., 2018). However, we hypothesize herein that it may also be possible to direct AMSCs toward the SSC phenotype through modulation of the cell microenvironment.

The effect of key physiological modulators on AMSC behavior was sequentially investigated through the control of environmental factors (i.e., collagen density and oxygen tension). The emergence of an SSC-associated transcriptional profile and markers of cell identity in response to their environment was also measured. Interestingly, concurrent shifts in cell status linked to transient exposure to distinct collagen densities were observed within the novel transitional culture system that was developed. We identified a shift toward early SSCs and late bone-cartilage-stromal progenitor (BCSP) status using previously published cell surface markers (PDPN, CD146, CD73, and CD164) when cells migrated from high (10% w/v) to low (0.2% w/v) collagen type I matrices under hypoxic conditions. Importantly, potency was enhanced when cultured in low (0.2%) collagen type I gels. This study uncovers a key synergy between collagen density and oxygen tension in regulating cellular behavior and indicates a means of isolating SSCs from AMSCs primarily through the modification of the cell’s microenvironment.

## Results

### 3D 0.2% collagen gels promote expression of stem cell and osteochondrogenic associated genes compared to cells cultured in 2D monolayers and 3D 10% collagen gels

**Figure 1:**
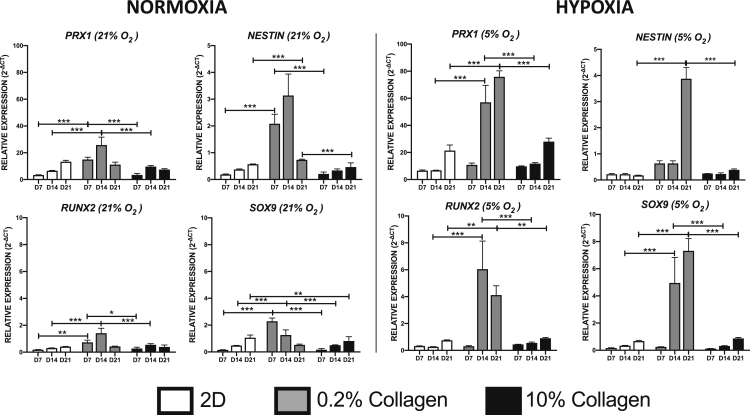
The effect of collagen density and oxygen tension on AMSC mesenchymal progenitor and osteochondrogenic gene expression AMSCs were cultured in either 2D (tissue culture plastic) or 3D (0.2% and 10%) collagen type I gels for a period of 7, 14, and 21 days in either normoxic (21% O_2_) or hypoxic (5% O_2_) conditions. Gene expression markers were assessed using qPCR (Data are presented as mean ±S.E.M, ssstatistical analysis performed using one-way analysis of variance, uncorrected Fisher’s least significant difference; ∗∗∗p < 0.001; ∗∗p < 0.01; ∗p < 0.05; n = 3).

Before assessing the biophysical and physiological parameters associated with the control of AMSC identity, the cells were evaluated for their differentiation potential. The differentiation of cell capacity toward chondrocytes, osteoblasts, and adipocytes was confirmed through the analysis of matrix deposition, mineralization, and lipid accumulation, respectively. In addition, gene expression analysis for factors defining cell phenotype correlated with this analysis (Figure S1). Cells were additionally assessed for their viability upon plastic compression (to achieve the 10% collagen constructs) before conducting any further studies. 75% of the cell population within the 10% collagen gels was deemed viable within 7 days of culture (Figure S2). Subsequently, the cells were assessed for the expression of mesenchymal stem cell markers, *PRX1 and NESTIN* (Méndez-Ferrer et al., 2010; Ouyang et al., 2014), in response to their biophysical environment (Figure 1). Under normoxic conditions, cells cultured within the 0.2% collagen gel exhibited a significantly higher expression profile of *PRX1 (*4.5-fold; p *<* 0.001) and *NESTIN* (11-fold; p *<* 0.001) as early as day 7, relative to 2D monolayers. Furthermore, a significantly higher expression of *PRX1* and *NESTIN* was observed in cells cultured in 0.2% collagen gels compared to those cultured within the 10% collagen gels at day 7 (*PRX1*: 4.3-fold, p *<* 0.001; *NESTIN*: 10-fold, p *<* 0.001).

A similar trend was observed with regards to the transcription factors conferring osteogenic and chondrogenic potential. Under normoxic conditions, a significantly higher expression of the osteogenic transcriptional marker, *RUNX2*, was observed in the 0.2% collagen gel compared to cells cultured in 2D monolayers (4-fold, p *<* 0.01) and 10% collagen gels (2.7-fold, p *<* 0.05) as early as day 7. In addition, a significant increase in the chondrogenic transcriptional marker *SOX9* was observed in 0.2% collagen gels compared to 2D monolayers (15.2-fold, p *<* 0.001) and 10% collagen gels (14.3-fold, p *<* 0.001) at day 7.

Under hypoxic conditions, a significantly higher expression was observed at day 14 in 0.2% gels with respect to *PRX1* (compared to 2D monolayer: 8.4, p *<* 0.001 and 10% collagen gel: 4.9, p *<* 0.001) and at day 21 with respect to *NESTIN* (compared to 2D monolayer: 22.8-fold, p *<* 0.001 and 10% collagen gel: 9.9-fold, p *<* 0.001). In addition, cells cultured in the 0.2% collagen gel expressed a higher level of *RUNX2* (compared to 2D monolayer: 22.2-fold, p *<* 0.001 and 10% collagen gel: 10.9-fold, p *<* 0.001) and *SOX9* (compared to 2D monolayer: 14.6-fold, p *<* 0.001 and 10% collagen gel: 15.5-fold, p *<* 0.001) at day 14 compared to other conditions. AMSCs cultured in 2D monolayers and 3D 10% collagen gel exhibited no significant induction in expression among all the analyzed genes over the time course of the experiment, irrespective of oxygen conditions. Importantly, the expression level of all genes was generally higher following 21 days of culture in the 0.2% collagen gels under hypoxia compared to the corresponding time point in any other experimental conditions.

### Transitional culture- the rescue of early progenitor status and identification of a human skeletal stem cell phenotype

**Figure 2:**
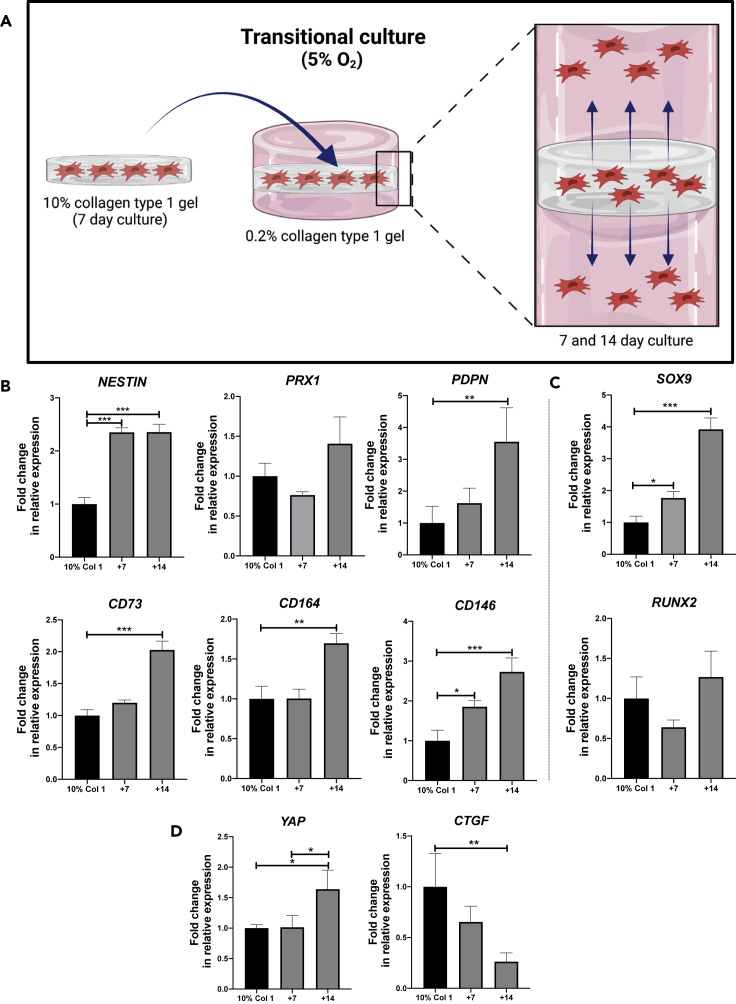
Development of a transitional culture to rescue AMSC skeletal stem cell phenotype (A) Schematic diagram illustrating the transitional culture. AMSCs were cultured in a 10% collagen type I gel for 7 days and subsequently introduced into a 0.2% collagen type I gel for a further 7 and 14 days. (B) Gene expression profile of progenitor cell markers were quantified using qPCR; human skeletal stem cell makers (CD164, PDPN, CD73), mesenchymal progenitor markers (NESTIN and PRX1), and a bone-cartilage skeletal progenitor marker (CD146); (C) Osteoblast and chondrocyte transcription factors RUNX2 and SOX9, respectively; and (D) Matrix stiffness associated markers (YAP, CTGF). (Data are presented as mean ±S.E.M, statistical analysis performed using one-way analysis of variance, uncorrected Fisher’s least significant difference; ∗∗∗p < 0.001; ∗∗p < 0.01; ∗p < 0.05; n = 3).

**Figure 3:**
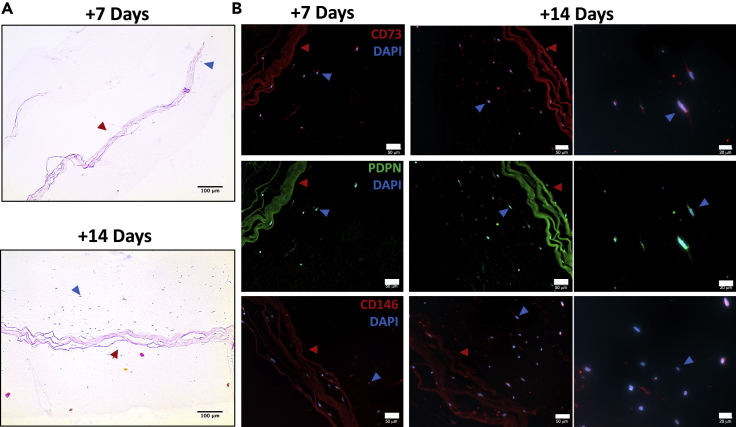
Migration and differential protein expression of AMSCs from a high density collagen gel to a low density collagen gel (A) Transitional cultures stained with H&E. Cells were visible in the 0.2% collagen scaffold by day 14 (scale bar: 100 μm). (B) Immunofluorescent staining of CD73, PDPN, and CD146 of the transitional cultures. Red arrows indicate the 10% collagen type I gel; blue arrows indicate cells migrated into 0.2% collagen type I gel (scale bar: 50 μm, 20 μm).

As previously demonstrated, AMSCs cultured within a 10% collagen gel exhibited low expression of all tested lineage markers under hypoxic conditions. However, culturing AMSCs in a 0.2% collagen gel exhibited significantly higher expression of these markers under hypoxic conditions (Figure 1). A transitional 3D model was therefore established to determine whether cells pre-cultured within a 10% collagen gel would migrate toward an environment conducive to reestablishing their early lineage/stem cell status (schematic diagram in Figure 2A). A hypoxic environment was chosen to encourage the aforementioned transcriptomic profile. Simultaneously, the cells were assessed for markers associated with early skeletal stem cells (*CD73* and *CD164*), bone-cartilage-stromal progenitors (*CD146*), and markers to identify the presence of a skeletal lineage population (podoplanin; *PDPN*). In addition, the cells were assessed for the osteochondrogenic transcription factors, *SOX9* and *RUNX2*, and for their response to a shift in matrix properties by measuring the expression of mechano-sensing YAP signaling molecules. AMSCs were cultured in a 10% collagen gel for 7 days and subsequently embedded between two 0.2% collagen gels and cultured for an additional 7 and 14 days (a time point previously indicative of a significant increase in progenitor gene expression profile). As shown in Figure 3A, early signs of cellular migration were observed by day 7, with an increase in cell numbers observed by day 14 (confirmed by the positive haematoxylin nuclear stain).

### Cells that migrated into the 0.2% collagen gel displayed a skeletal stem cell profile under hypoxic conditions

Upon nesting the seeded 10% collagen gel between the 0.2% collagen gels to form the transitional culture, gene expression analysis of the transitional culture in its entirety was measured 7 and 14 days post-embedding. A significant 2-fold increase (p *<* 0.001) in *NESTIN* expression relative to the cells cultured in the 10% collagen gel alone was observed 7 days post-embedding (Figure 2B), which persisted through day 14. A trend toward an increase in *PRX1* expression was observed following 14 days. Interestingly, a significant 3.5-fold increase in *PDPN* expression was observed by day 14 (p *<* 0.01) relative to the cells cultured within the 10% collagen gel. With regards to early skeletal stem cell markers, a significant 2-fold increase (p *<* 0.001) in *CD73* and a 1.7-fold increase (p *<* 0.01) in *CD164* expression were observed 14 days after embedding, compared to the cells cultured within the 10% collagen gel. A significant 1.8-fold increase (p *<* 0.05) in the bone-cartilage-stromal marker *CD146* was observed 7 days post-embedding, with a 2.7-fold increase (p *<* 0.001) in expression observed by day 14 compared to cells cultured within the 10% collagen gel before embedding.

The cells were additionally assessed for their osteochondrogenic marker profile (Figure 2C). A significant (1.8-fold, p *<* 0.05) increase in *SOX9* expression was observed in the transitional culture by day 7, followed by a 3.9-fold (p *<* 0.001) increase by day 14 compared to cells cultured within the 10% collagen gel alone. No significant change in *RUNX2* expression was observed. Furthermore, the cells were assessed for their response to a shift in collagen densities (Figure 2D). The expression of *YAP* was significantly increased by 1.4-fold at day 14 in the cells cultured within the transitional culture compared to cells cultured within the 10% collagen gel. Interestingly, the expression of *CTGF* (a canonical downstream target of YAP/TAZ signaling) was significantly decreased by 3-fold at day 14 in the transitional culture compared to the cells cultured within the 10% collagen gel (p *<* 0.01).

Immunofluorescent staining for PDPN, CD73, and CD146 confirmed differential expression of these markers among the cells within the transitional culture (Figure 3B). Cells that migrated into the 0.2% collagen gel exhibited PDPN positivity with limited expression visible within the 10% collagen gel (Figure 3B). CD73 was primarily expressed in cells that migrated into the 0.2% collagen gel with minimal CD146 positivity detected. Static cells that remained within the 10% collagen gel, however, were CD146+ with limited CD73 detected. Exposing the cells to the 0.2% collagen gel therefore promoted an early skeletal progenitor phenotype; with cells residing in the 10% collagen gel by day 14, maintaining a mature bone-cartilage-stromal phenotype is suggested by a positive CD146 protein expression.

### Migration of cells toward the 0.2% collagen gel reprograms cells to an SSC profile, with cells retained within the 10% collagen gel favoring a transitioned bone-cartilage-stroma progenitor under hypoxic conditions

**Figure 4:**
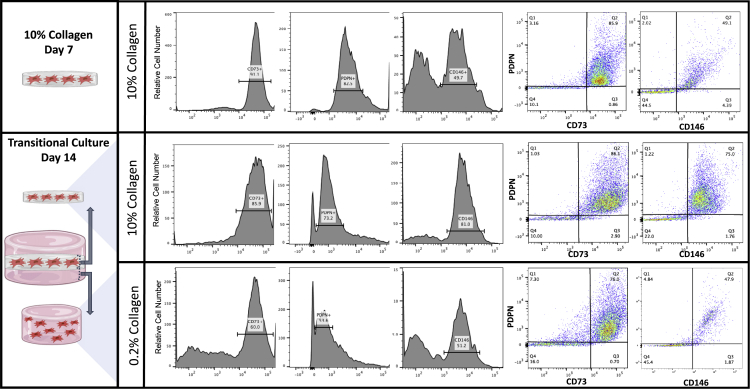
Cell surface analysis of AMSCs within the transitional culture AMSCs were cultured in a 10% collagen type I gel for 7 days and embedded in a 0.2% collagen type I gel, making up the transitional culture for an additional 14 days. Flow cytometric analysis was conducted for PDPN, CD73, and CD146 (representative data presented).

Assessing the cell’s transcriptional profile as described provides an indication of the overall status of the culture when exposed to specific culture conditions. However, to further determine the identity of the cells responsible for this transcriptomic shift, flow cytometric analysis was conducted for the early skeletal stem cell marker (CD73) and a bone-cartilage skeletal marker (CD146). PDPN was examined on all cell populations to ensure the presence of a skeletal stem lineage cell (Figure 4). Flow cytometry was conducted on cells isolated from the 10% collagen gel at day 7 (before embedding within the transitional culture) and 14 days after embedding within the transitional culture. In addition, the cells present within the 0.2% collagen gel alone were assessed on day 14 (Figure 4). Isolated cells were PDPN+ throughout the culture, confirming the presence of a skeletal stem lineage cell population. Before fabricating the transitional culture, cells present within the 10% collagen gel at day 7 exhibited a population enriched for CD73^+^ cells (representative of a human skeletal stem cell phenotype; 85.9% positivity) relative to CD146 (representative of a bone cartilage stroma phenotype; 49.1% positivity). Upon embedding, static cells that remained within the 10% collagen gel were maintained as a heterogeneous population, exhibiting a 25.9% increase in CD146+ (75%) cells compared to day 7 and no difference in CD73^+^ cells (86.1%). Cells that migrated into the 0.2% collagen gel contained a population similar to a human skeletal stem cell, rich in CD73 (represented by 76% of the population) compared to a CD146 (45.5%) at day 14. The migration of cells toward the 0.2% collagen gel represented an early skeletal stem cell phenotype compared to the transitioned BCSP population of cells enriched within the 10% collagen gel.

### AMSCs cultured in 0.2% collagen gels under hypoxic conditions exhibit an enhanced SSC potency compared to conventional 2D culture conditions

To further validate the presence of an SSC profile of AMSCs detected within the 0.2% collagen gels under hypoxic conditions, AMSCs were analyzed for their differentiation potential, a key characteristic of an SSC phenotype. Briefly, AMSCs were precultured in either a 2D monolayer or a 0.2% collagen gel for a period of 14 days under hypoxic conditions. The gels were digested and a chondrogenic, osteogenic, and adipogenic differentiation assay was conducted on both cell populations.

**Figure 5:**
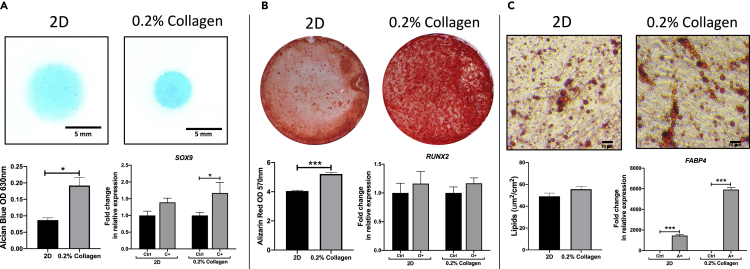
AMSC potency in response to preconditioning in a 0.2% collagen gel and 2D monolayer at hypoxic oxygen tension (A) Chondrogenic micromasses were stained with Alcian Blue to detect sulfated GAGs (Top, scale bar = 5 mm) after 7 days and SOX9 expression analyzed by qPCR (Bottom). (B) An osteogenic differentiation assay was conducted for 21 days, illustrating calcium phosphate staining using Alizarin Red (Top) and gene expression of osteogenic associated transcription factor RUNX2 analyzed by qPCR (Bottom). (C) An adipogenic differentiation assay was cultured over 21 days with the presence of fat droplets analyzed using Oil Red O stain (Top, scale bar = 10 μm) and FABP4 gene expression analyzed (Bottom). (Data are presented as the mean ±S.E.M, statistical analysis performed using Mann-Whitney test; ∗∗p < 0.01; ∗p < 0.05; n = 3).

Cells precultured in 0.2% collagen gels under hypoxic conditions exhibited an enhanced chondrogenic potential (Figure 5A). This is indicated by the significantly higher Alcian Blue staining (indicative of GAG deposition and therefore chondrogenic differentiation) detected from the cells precultured in the 0.2% collagen gels (p *<* 0.05) compared to 2D monolayers. In agreement, a significantly higher expression of *SOX9* was observed in the chondrogenic induced condition compared to control in cells precultured in the 0.2% collagen gels *(*p *<* 0.05). However, no difference in *SOX9* expression was detected in the chondrogenic induced condition compared to control in 2D monolayers.

Cells precultured in 0.2% collagen gels under hypoxic conditions exhibited an enhanced osteogenic potential. Indeed, a significantly higher Alizarin Red stain (representative of mineral deposition and indicative of osteogenic differentiation) was observed in cells precultured in the 0.2% collagen gels relative to cells precultured in 2D monolayers (p *<* 0.001) (Figure 5B). Interestingly, no significant differences were observed in the expression of the osteogenic transcription marker, *RUNX2,* between the induced and control samples in both precultured conditions.

With regards to the cell’s adipogenic potential, positive Oil Red O stain indicative of lipid droplets were observed in both conditions (ns). A significant increase in *FABP4* gene expression (p *<* 0.01) was observed in the adipogenic induced samples compared to control in both precultured conditions (Figure 5C).

### Rheological characteristics of a SSC promoting environment

**Figure 6:**
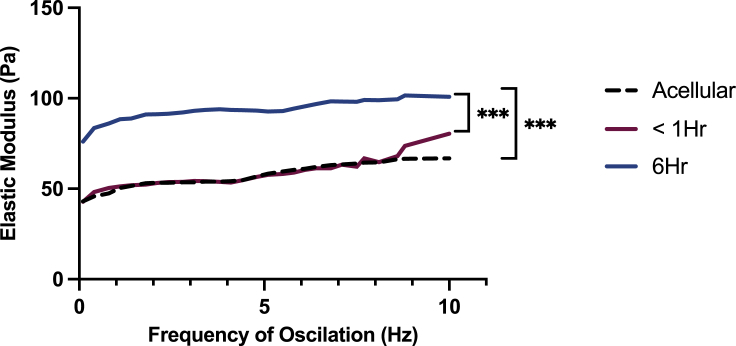
Rheological characterization of the SSC promoting 0.2% collagen type I gel Stiffness measurements of the 0.2% collagen type I gel with and without AMSCs. Stiffness measurements conducted on acellular gels (black dotted line) and cellular gels within 1 hour of seeding (Red) and 6 hours post seeding (Blue) (Statistical analysis performed using one-way analysis of variance, uncorrected Fisher’s least significant difference; ∗∗∗p < 0.001; n = 3).

To further characterize the 0.2% collagen gel as a platform for directing an SSC identity of AMSCs, the gel was measured for its rheological properties to provide a stiffness measurement as a means of correlating the effect of collagen density and material stiffness on AMSC behavior. In addition, the presence of AMSCs to the gel and their effect on the rheological properties of the matrices were also measured. 0.2% collagen gels exhibited a Young’s Elastic modulus ranging between 43 to 70 Pa. The addition of cells did not significantly alter the gels resistance to deformation within an hour of seeding, exhibiting Youngs’ Elastic moduli measurements ranging between 42.78 and 80 Pa. However, 6 hours after cell seeding, a significant increase in resistance to matrix deformation was detected compared to acellular 0.2% collagen gels (p < 0.001) and gels within an hour of seeding (p < 0.001), exhibiting measurements ranging between 76 and 100 Pa (Figure 6).

## Discussion

Through the adoption of a reductionist approach and by deconstructing the complex *in vivo* cell environment, we are beginning to understand the tremendous impact of environmental factors, including mechanical forces, in regulating cell behavior. Although it has been reported that variations in collagen density can regulate cell behavior (Trappmann et al., 2012; Joshi et al., 2017), synergies with the effect of oxygen tension have not yet been investigated thoroughly. Herein, we show that culturing AMSCs within 0.2% collagen gels under hypoxic conditions results in the emergence of a skeletal stem cell profile. As these cells migrate between 10% and 0.2% collagen matrices, they alter the expression of early skeletal stem cell markers and lineally transitioned mature markers of their progeny. Fundamentally, this provides a novel approach to trigger skeletal reprogramming of human AMSCs.

Multiple studies have documented the effect of environmental stresses on modulating MSC gene expression profile, specifically regulating early fate decisions (Anderson and Knothe Tate, 2007; McBride et al., 2008; Knothe, 2011). Furthermore, different cell types have distinct metabolic demands, each demonstrating alternate oxygen consumption rates required to retain cellular potency. Culturing AMSCs under hypoxic conditions within a 0.2% collagen gel resulted in the highest expression of all progenitor and osteochondrogenic markers by day 14. It is important to note that the collagen gels used in this study allow for successful oxygen permeation (Cheema et al., 2011; Streeter and Cheema, 2011). Thus, the local oxygen tension to which the cells are exposed to is relatively proportional to the applied oxygen tension (Cheema et al., 2011).

It has previously been reported that 3D cultures mimicking the bone marrow MSC environment demonstrated enhanced stem cell potency at 21% O_2_ (Winer et al., 2009). However, this study suggests solely a transient maintenance of MSC potency when equivalent conditions are applied. Providing an oxygen tension representative of physiological oxygen conditions (5% O_2_), however, resulted in the delayed onset, but long-term maintenance of a significantly higher expression of the early lineage markers was examined. It is important to note that the oxygen consumption rate of MSCs within these collagen matrices upon exposure to low oxygen tensions has been previously established, measuring an average rate constant of 7.91 × 10^−18^ mol cell^−1^ s^−1^ (Streeter and Cheema, 2011). The established value represents the rate of oxygen consumption as measured *in vivo*. This low oxygen consumption rate has been proposed to promote a quiescent stem cell phenotype. Importantly, the rate of oxygen consumption is reported to be much lower than that exhibited by cells cultured in 2D environments. This highlights the significance in studying cell behavior in 3D environments with adequate perfusive properties, mimicking both the geometric/ ECM of a cell as well as an environment allowing sufficient nutrient supply to closely mimic *in vivo* cell behavior (Streeter and Cheema, 2011).

Pretreatment of AMSCs under these favorable conditions (5% O_2_) for 14 days further enhanced the cell’s potency for osteogenic and chondrogenic differentiation when transitioned to ambient oxygen and conventional 2D culture methodologies. Similarly, a previous study conducted by Inagaki et al. (2017) showed that pretreating rat bone MSCs at 5% O_2_ for 7 days significantly enhanced the cell’s osteogenic potential when transitioned to ambient oxygen conditions, compared to cells pretreated in 21% O_2_ (Inagaki et al., 2017). In addition, a study carried out by Pfeiffenberger et al. (2019) showed enhanced equine MSC osteogenic potential when pretreated in oxygen conditions as low as 1% (Pfeiffenberger et al., 2019). In line with the methods used by Inagaki et al., (2017), our study suggests that pretreating MSCs under hypoxic conditions augmented the cell’s osteogenic and chondrogenic differentiation capacity upon chemical stimulation within a normoxic environment (Inagaki et al., 2017). This finding suggests that culturing MSCs within oxygen tensions mimicking their native environment will enhance the cell’s potency relative to conventional culture methodologies. Furthermore, these results show that the physiological oxygen-associated cellular activation is not transient and these effects were adequately maintained within the collagen hydrogel system, confirming it as a tool for modulating stem cell potency.

The intracellular response triggered by the mechanical stimuli of the cell environment is known to be directed by the transcriptional regulators, Yes-associated protein (YAP)-transcriptional coactivator with PDZ-binding motif (TAZ) signaling. These regulate stem cell mechanical memory and act as an intracellular ‘mechanical rheostat’ to store information associated with past physical environments (albeit time-dependent), influencing cell fate (Lian et al., 2010; Caliari et al., 2016). To further investigate the influence of this hydrogel system as a tool for cellular expansion of relevant stem cell populations, pre-culturing AMSCs in the distinct environments (2D and 0.2% collagen gels) impacted the cells ability to differentiate and the degree of differentiation. These findings allude to a potential biological memory exhibited by MSCs, storing information from their physical environment, allowing this mechanical history to influence their future fate (Caliari et al., 2016).

In addition, the coordinated activation of these transcriptional regulators has emerged as a key mediator of osteoblast progenitor proliferation and has enhanced capacity for differentiation during bone development (Caliari et al., 2016; Kegelman et al., 2018; Pan et al., 2018; Xiong et al., 2018). Exposing AMSCs to the transitional culture in our study resulted in a significant increase in *YAP* gene expression upon exposure of the cells to the low collagen environment. This finding interestingly coincides with that of embryonic stem cells exhibiting elevated YAP/TAZ activity, which in turn plays a key role in their self-renewal and maintenance of a stem cell phenotype (Lian et al., 2010). In addition, Kegelman et al. (2020) demonstrated the importance of YAP/TAZ expression in promoting periosteal progenitor expansion and osteoblastic differentiation to mediate bone fracture repair (Tang et al., 2016). YAP signaling induced by the transitional culture is supported by the increased expression of all skeletal progenitor markers measured. Conversely, a significant decrease in the downstream stiffness associated with marker *CTGF* was demonstrated over the 14-day culture in a low collagen environment. Although CTGF expression is associated with the presence of a stiff environment, it is synergistically expressed during stem cell differentiation along the osteochondrogenic lineage (Tang et al., 2016; Kegelman et al., 2020). This expression profile further supports the establishment of an early stem cell profile with respect to the cells that migrated toward the lower collagen density environment.

Correlating with the increase in YAP signaling, the migration of AMSCs toward the low collagen matrix within the transitional culture system resulted in the presence of an early skeletal progenitor population over 14-days with a significant increase in *CD164* and *CD73*. When subjected to flow cytometry, the cells were predominantly PDPN^+^CD73^*+*^ with minimal *CD146* expression, representative of an undifferentiated hSSC population. In line with these findings, Chan et al. (2018) reported the discovery of a similar human cell population with an equivalent surface protein expression profile by stimulating adipose tissue with recombinant BMP2 (hSSC: PDPN^+^CD146^-^CD73^+^CD164^+^) (Chan et al., 2018). In comparison, our study achieves a SSC phenotype of AMSCs through the manipulation of the cell microenvironment and oxygen tension. This study, therefore, identifies a chemically-independent methodology to obtain this phenotype (Wenger et al., 2007; Chan et al., 2018; Al Hosni et al., 2020).

To further develop our knowledge on the specific properties required to induce this SSC phenotype, the gel was measured for its rheological properties. The Young’s Elastic moduli of 80–100 Pa was observed in the presence of cells. This is a key factor to account for when creating larger constructs for cellular expansion. Subject to cell size and spreading, cell density should be optimized accordingly to avoid any further deformation of the construct, with the potential of influencing cellular behavior. Our transitional culture implies that a cellular density of 22,560 cells/cm^3^ is an appropriate number of AMSCs to be cultured within a 0.2% collagen gel to maintain optimum culture conditions throughout the construct.

To put the rheological properties of this construct into perspective with regards to their effect on cellular behavior, a previous study conducted by Winer et al. (2009) reported the successful maintenance of bone marrow MSC self-renewal and potency when cultured in a polyacrylamide gel with a similar stiffness measurement of 220 ± 50 Pa, compared to cells grown on stiffer substrates with a stiffness measurement above 7500 Pa (Winer et al., 2009). Both studies suggest that soft matrices can maintain MSCs in an early cell state while preserving their differentiation capacity. Importantly, this finding is simultaneously attributed to the presence of a hypoxic environment.

In conclusion, to our knowledge, the transitional culture presented here is the first report of the influence of collagen density and oxygen tension in regulating the developmental hierarchy of stem cells, with AMSCs displaying a SSC signature in a 0.2% collagen gel and a transitioned early BCSP when exposed to a 10% collagen gel. This breakthrough enhances our understanding of the intrinsic mechanisms involved in regulating stem cell behavior to develop methodologies to reprogram and select for SSC *in vitro* without the requirement of exogenous growth factors or chemicals. This has the advantage of being immediately scalable, which is a critical step in tissue regeneration strategies. Furthermore, this transitional culture system is an effective tool for the investigation on how cell populations interact with their environment to regulate their state. These findings have significant implications in developing methods to isolate and expand specific cell types from MSC populations. This study sheds light on the impact of conventional culture conditions on cell characteristics *in vitro* and the need to develop more relevant culture systems for the maintenance of stem cell behavior.

### Limitations of the study

It is important to note that although the presented study provides a novel approach to reprogramming stem cell phenotypes, there are some limitations that must be addressed. For example, although we used immortalized AMSCs as a homogeneous, robust, and reproducible cell population to delineate the effect of collagen density and oxygen tension, these data should be confirmed with multiple primary human counterparts. However, this is beyond the scope of the current study as the inherent heterogeneity of these populations would have made the assessment of biophysical and physiological cues problematic. Furthermore, despite the use of collagen density as a surrogate for the microenvironment that the cells find themselves in, it is likely that porosity and pore size will also change. These will also likely have pronounced effects on cell behavior. However, measuring accurately and quantifying in this type of matrix is technically challenging. Future work should aim to further characterize these aspects of the scaffold to determine their effect. Nevertheless, the diffusion coefficients of both oxygen and glucose have previously been determined, which are factors that can be directly impacted by porosity (Rong et al., 2006, Cheema et al., 2011). Cell migration through the scaffolds has also been reported (Hadjipanayi et al., 2010). Lastly, rheological properties of the 10% collagen gels were not measured because of their geometry. Previous studies have optimized a method including atomic force microscopy (AFM) to successfully measure the stiffness of 10% collagen gels (Al Hosni et al., 2020; Schuh et al., 2021). The use of AFM, however, could not be applied to the 0.2% collagen gel, as locating individual collagen fibrils and landing the probe was not feasible in the hydrated state of the gel, unless the gels were subjected to dehydration, impacting fibril diameter. As a result, two comparable methodologies would be required to measure the stiffness properties of the 0.2% collagen gel and 10% collagen gel using rheology and AFM, respectively. Thus, a focus was directed on the 0.2% collagen gel to further characterize its physical properties as a tool for studying stem cell behavior.

## STAR★Methods

### Key resources table

**Table:** 

REAGENT or RESOURCE	SOURCE	IDENTIFIER
**Antibodies**		

Recombinant Anti-CD73 antibody [EPR6114]	Abcam, Cambridge, UK	Abcam Cat# ab124725, RRID:AB_10976033
Anti-CD164 (H-4) monoclonal antibody	Santa Cruz Biotechnology, TX, USA	Santa Cruz Biotechnology Cat# sc-271179, RRID:AB_10613973
Anti-PDPN (B-11) monoclonal antibody	Santa Cruz Biotechnology, TX, USA	Santa Cruz Biotechnology Cat# sc-166906, RRID:AB_10610482
VectaShield Antifade mounting medium containing DAPI	Vector Laboratories, Burlingame, CA, USA	Vector Laboratories Cat# H-1200, RRID:AB_2336790
Podoplanin Monoclonal Antibody (NZ-1.3), PE, eBioscience™	Thermo Fisher Scientific, Loughborough, Leicestershire, UK	Thermo Fisher Scientific Cat# 12-9381-41, RRID:AB_1582263
CD73 Monoclonal Antibody (AD2), FITC, eBioscience™	Thermo Fisher Scientific, Loughborough, Leicestershire, UK	Thermo Fisher Scientific Cat# 11-0739-42, RRID:AB_10596508
CD146 Monoclonal Antibody (P1H12), FITC, eBioscience™	Thermo Fisher Scientific, Loughborough, Leicestershire, UK	Thermo Fisher Scientific Cat# 11-1469-42, RRID:AB_2043805

**Chemicals, peptides, and recombinant proteins**		

Recombinant Human TGF-β1 (HEK293 derived)	PeproTech, London, UK	Cat#100-21
Dexamethasone	Sigma, UK	Cat#D4902
L-Proline	Sigma, UK	Cat#P5607
L-ascorbic acid 2-phosphate	Sigma, UK	Cat#A8960
3-Isobutyl-1-methylxanthine	Sigma, UK	Cat#5879
Sodium hydroxide	Sigma, UK	Cat#S5881
β-Glycerophosphate disodium salt hydrate	Sigma, UK	Cat#G9422
Rat-tail collagen type I (2.05 mg/ml in 0.6% acetic acid)	First Link (Ltd), UK	Cat#60-30-810

**Critical commercial assays**		

RNeasy Mini kit	Qiagen, Manchester, UK	Cat#74106
High-Capacity cDNA Reverse Transcriptase Kit	Thermo Fisher Scientific, Loughborough, Leicestershire, UK	Cat#368814
iTaq Universal SYBR Green Super mix	Bio-Rad, Hertfordshire, UK	Cat#1725124
LIVE/DEAD™ reagents	Theermo Fisher Scientific, Loughborough, Leicestershire, UK	Cat#L-3224
Real Architecture For 3D Tissues (RAFT™)	Lonza Bioscience	Cat#016-1R16

**Experimental models: Cell lines**		

hTERT-immortalised human (female) AMSCs	ATCC, MNZ, VA, USA	ATCC Cat# SCRC-4000,RRID:CVCLU602, MNZ

**Oligonucleotides**		

Primers for *CD146* Forward: GGAAGGT GTGGGTGAAAGAG	This paper	N/A
Primers for *CD146* Reverse: GGACATTC AGGGTGCTCAG	This paper	N/A
Primers for *CD164* Forward: CCTTAGCT TTCTCCCGAACG	This paper	N/A
Primers for *CD164* Reverse: TGCTGGG TCGTGTTCTTG	This paper	N/A
Primers for *CD73* Forward: ACTGGGAC ATTCGGGTTTTG	This paper	N/A
Primers for *CD73* Reverse: CTCTTTGGA AGGTGGATTGC	This paper	N/A
Primers for *FABP4* Forward: CATACTG GGCCAGGAATTTG	This paper	N/A
Primers for *FABP4* Reverse: GGACAC CCCCTCTAAGGTT	This paper	N/A
Primers for *GAPDH, NES, PDPN, PRX1, RUNX2, SOX9, TAZ, YAP,* see Table S1	This paper	N/A

**Software and algorithms**		

FlowJo™ software	FlowJo, LLC	FlowJo, RRID:SCR_008520
ImageJ	ImageJ	National Institute of Health, RRID:SCR_003070 (https://imagej.nih.gov/ij/)
GraphPad Prism version 6.0f for windows	GraphPad Prism Software, La Jolla, CA, USA	http://www.graphpad.com

### Resource availability

#### Lead contact

Further information and requests for resources and reagents should be directed to and will be fulfilled by the lead contact, Scott J. Roberts (sjroberts@rvc.ac.uk).

#### Materials availability

This study did not generate new unique reagents.

### Experimental model and subject details

#### Human immortalised AMSC culture

hTERT-immortalised human (female) AMSCs were obtained from ATCC (ATCC Cat# SCRC-4000, RRID:CVCL_U602, MNZ, VA, USA) and cultured in growth medium composed of high glucose Dulbecco’s Modified Eagle’s Medium (DMEM, ThermoFisher Scientific, Loughborough, Leicestershire, UK) supplemented with 10% batch-tested foetal bovine serum (FBS) and antibiotics-antimycotic solution (100 units/mL penicillin, 100 μg/mL streptomycin and 0.25 μg/mL amphotericin B; ThermoFisher Invitrogen, Paisley, UK). AMSCs were expanded in 37°C, 5% CO_2_/air and 95% humidity. All experiments described herein were carried out between passages 6–7. Cells were cultured in T225 flasks for expansion.

### Method details

#### Characterisation of AMSCs

AMSCs were expanded in growth medium to 70% confluency and subjected to adipogenic, osteogenic and chondrogenic differentiation to assess for their trilineage potential.

Osteogenic differentiation was assessed by seeding cells at a density of 4,500 cells/cm^2^ on a tissue culture dish in normal growth medium. After 24 h, freshly prepared osteogenic media composed of growth medium supplemented with 100 nM dexamethasone (Sigma, Gillingham, Dorset, UK), 50 μg/mL L-ascorbic acid 2-phosphate (Sigma) and 10 mM β-glycerophosphate (Sigma) was prepared and cells cultured in these differentiating conditions for 21 days. Controls in growth medium were cultured in parallel to the osteogenic differentiation conditions, with media changes occurring every 3 days. Differentiation was assessed by staining calcium mineral deposition with alizarin red solution (pH 4.2). Additionally, RNA was extracted and gene expression of *RUNX2* quantified using SYBR green qPCR.

Chondrogenic differentiation was assessed by seeding AMSCs in micro masses with a cell density of 5,000 cells/μL in growth medium. Cells were allowed to adhere for 2 h before culturing in a chondrogenic medium consisting of low glucose DMEM (ThermoFisher Invitrogen), 1x insulin-transferrin-selenium supplement (Corning, Wiebaden, Germany), dexamethasone (100 nM, Sigma), Y27632 (10 μM, Axon Medchem, Groningen, Netherlands), L-ascorbic acid 2-phosphate (50 μg/mL, Sigma), L-proline (40 μg/mL, Sigma) and transforming growth factor-β1 (TGF- β1; 10 ng/mL, PeproTech, London, UK) for 7 days. Differentiation was evaluated by staining glycosaminoglycan (GAG) deposition with Alcian Blue dye (Sigma) (pH 2.0) overnight at room temperature. Additionally, RNA was extracted and gene expression of *SOX9* quantified using SYBR green qPCR.

Adipogenic differentiation was assessed by seeding AMSCs at a cell density of 30,000 cells/well in a 48 well plate for a period of 21 days. The cells were seeded in growth medium and allowed to reach confluency. Once confluency was reached the media was substituted with growth medium, insulin (1 μg/mL, Sigma), dexamethasone (0.1 μM, Sigma), 3-Isobutyl-1-methylxanthine (IBMX; 4.5 μM, Sigma) and indomethacin (125 μM, Sigma), which was refreshed every 2 days. Production of fat droplets was assessed using Oil Red O stain. Additionally, RNA was extracted and gene expression of *FABP4* quantified using SYBR green qPCR.

#### Fabrication of collagen type I AMSC seeded gels

Collagen type I gels were prepared in accordance with the RAFT™ 3D cell culture protocol (Lonza Bioscience, Basel, Switzerland). A collagen master mix was prepared on ice, containing 10% 10x MEM (ThermoFisher) (used as a pH/colour indicator), 80% rat-tail collagen type I (2.05 mg/mL in 0.6% acetic acid, First Link, Birmingham, UK), 6% neutralizing agent (840 mM HEPES buffer (ThermoFisher) and 1.65 M NaOH (Sigma) to obtain a pH of 7.4 (Magdeldin et al., 2017). The 96% volume of neutralised collagen master mix was allowed to rest for a period of 30 min on ice to ensure the expulsion of air bubbles from the mixture and prevent gelation of the neutralised solution. At 80% cell confluency, a desired AMSC concentration of 30,000 cells/gel was prepared in growth medium at a final volume representative of 4% of the neutralised collagen master mix. The 4% cell suspension was gently pipetted into the collagen master mix and mixed to produce a homogenous cell-collagen solution, avoiding the production of air bubbles. 1.3 mL of the collagen master mix was dispensed into each well of a 24-well plate and allowed to gelate at 37°C for 15 min.

Upon gelation, a collagen hydrogel containing a large excess fluid to collagen ratio (0.2% w/v collagen type I) was formed. To achieve a denser collagen matrix composed of 10% w/v collagen type I, the hydrogels underwent plastic compression (controlled fluid removal process) using a hydrophilic Real Architecture For 3D Tissues (RAFT™) (Lonza Bioscience) absorber placed on top of the hydrogel for 15 min at room temperature. This system allows for rapid expulsion of fluid from the hydrogel to increase the density of the collagen matrix by 50-fold (Brown et al., 2005; Magdeldin et al., 2017; Kayal et al., 2019). The absorber was subsequently removed, and 1 mL of growth medium dispensed over the 10% collagen type I gel. Collagen gels retained as a 0.2% collagen type I gel were dispensed with 1 mL of growth medium. The gels were allowed to incubate for a period of 7, 14 and 21 days at 37°C, 5% CO_2_/air at either 5% O_2_ (hypoxia) or 21% O_2_ (normoxia) (provided through cell culture incubators) to assess the effects of oxygen tension and cell-extracellular matrix interactions on AMSC characteristics.

#### Rheological testing of 0.2% collagen type I gel

0.2% collagen type I gels (n = 3) were fabricated using the aforementioned technique. A 40 mm wide gel was fabricated to align with the 40 mm conical plate of the rheometer. Oscillatory measurements were performed on a Bohlin CVO rotational rheometer using a parallel plate geometry of 40 mm diameter with a measurement gap of 150 μm. The frequency of oscillation was set to a standardized range of 1–10 Hz (Fernández Farrés and Norton, 2014) with a constant strain of 0.2 Pa (maximum strain that can be applied at which the hydrogel is within its linear viscoelastic range). Material deformation was recorded over a period of 3 min at 37°C to avoid gel disruption. The elastic modulus of the matrix was measured with and without cells.

#### LIVE/DEAD™ cell viability assay

The LIVE/DEAD™ reagents (ThermoFisher Cat# L-3224) were used to determine the viability of AMSCs when subjected to plastic compression. AMSCs were seeded in 0.2% collagen type I gels and compressed using a RAFT™ (Lonza Bioscience) absorber to form a 10% collagen type I gel. Cell viability was measured immediately after compression (day 0) and after 7 days. LIVE/DEAD™ reagents, Calcein AM (4 mM in anhydrous DMSO) and Ethidium homodimer-1 (EthD-1, 2 mM in DMSO/H2O 1:4 (v/v)) were warmed to room temperature and used in the dark. 5 μL of Calcein-AM and 20 μL of EthD-1 was added to 10 mL of PBS to create a working solution. This was added to each well containing the hydrogel-cell constructs and incubated in the dark at 37°C for 1 h. The sample was then washed with PBS and imaged with an Axiovert200M microscope (Zeiss, Germany). Fluorescence intensity was quantified using ImageJ (National Institute of Health, RRID:SCR_003070).

#### Fabrication of transitional 3D culture

To define whether the AMSCs could alter cell phenotype with respect to matrix stiffness, the cells were cultured in 10% collagen type I gels at a cell density of 30,000 cells/gel for a period of 7 days under hypoxic conditions. The gels were further embedded between two 0.2% collagen type I gels in a 24-well plate. This was performed by preparing a 0.2% collagen gel and pipetting half the volume into a well and allowing it to gelate. The cellular 10% collagen gel was then carefully transferred onto the gelated 0.2% gel. The remaining half of the 0.2% gel was pipetted over to create a sandwich. The cultures were then incubated for a further 7 and 14 days under hypoxic conditions. To date, the diffusion coefficient of oxygen within the 10% collagen has been recorded at 4.5 × 10^−6^ cm^2^/s using Fick’s law model mimicking values represented by native fascia tissue (Cheema *et a*l., 2011). As described by Cheema et al. (2011), the lower the collagen density within a 3D gel the higher the rate of oxygen diffusion, thus, shedding light on a corresponding diffusion coefficient of oxygen within the 0.2% collagen gel. It is important to note that equal distribution of oxygen throughout the collagen gels has previously been shown, with the initial oxygen tension within these collagen gels equilibrating to the oxygen tension supplied from the external medium (Cheema et al., 2011). Gene expression profile of AMSCs was analysed using qPCR (described below) for early (*CD164, CD73, PDPN*) and late (*CD146*) lineage markers associated with the human skeletal stem cell. Additionally, the mesenchymal markers *NESTIN, PRX1, RUNX2* and *SOX*9 were quantified. Stiffness associated markers *YAP* and *CTGF* gene expression were quantified using SYBR green qPCR. Gels were fixed, paraffin-embedded, 5 μm sections prepared, and stained with haematoxylin and eosin.

#### Digestion of 0.2% collagen type I gels

AMSCs were seeded in 0.2% collagen type I gels for a period of 14 days under hypoxic (5% O_2_) conditions. Gels were digested in 200U Collagenase I (Sigma) for 1 h at 37°C. The cells were resuspended in growth medium and passed through a cell strainer to eliminate any debris and allow for a single cell suspension. The cells were subjected to an adipogenic, osteogenic and chondrogenic differentiation assay.

#### Quantitative PCR

Total RNA was isolated using the RNeasy Mini kit (Qiagen, Cat# 74106, Manchester, UK) following the manufacturer’s instructions. RNA was quantified using a NanoDrop spectrophotometer measuring at 260/280 nm, and 1 μg RNA/sample reverse transcribed using the High-Capacity cDNA Reverse Transcriptase Kit (ThermoFisher Scientific, Cat# 4368814) with the program: 25°C for 10 min, 37°C for 120 min, 85°C for 5 min, and infinite hold at 4°C. Transcribed cDNA was assessed using the CFX96 Touch™ Real-Time PCR Detection System (40 cycles) with the iTaq Universal SYBR Green Super mix (Bio-Rad, Cat#1725124, Hertfordshire, UK). Primers were designed using Primer3 and were selected to span an intron to isolate RNA specific amplification. Relative differences in expression were calculated using 2^-ΔΔCt^ (Livak and Schmittgen, 2001) and normalised to *GAPDH* expression. Primer sequences can be found in Table S1.

#### Immunofluorescent staining

Transitional 3D gels were fixed in 10% formalin for 1 h. Gels were paraffin embedded and sectioned. Samples were deparaffinised in xylene and re-hydrated in 95 and 70% absolute alcohol. Antigen retrieval was performed using 10 mM sodium citrate buffer (pH 6) at 95°C for 5 min. Samples were blocked using 1% (w/v) bovine serum albumin (BSA, Sigma) in 0.3% Triton X-100 in H_2_0 for 1 h at room temperature. Anti-CD73 monoclonal antibody (Abcam Cat# ab124725, RRID:AB_10976033, Cambridge, UK), anti-CD164 monoclonal antibody (Santa Cruz Biotechnology Cat# sc-271179, RRID:AB_10613973, TX, USA) and anti-PDPN monoclonal antibody (Santa Cruz Biotechnology Cat# sc-166906, RRID:AB_10610482) were diluted 1:200 in blocking buffer and incubated with the section for 1 h at room temperature. Appropriate secondary antibodies were further stained at a dilution of 1:500 in blocking buffer and added to the section for a period of 2.5 h at room temperature. Cells were counterstained with VectaShield Antifade mounting medium containing DAPI (Vector Laboratories Cat# H-1200, RRID:AB_2336790, Burlingame, CA, USA). The fluorescent signal was imaged on an Axiovert200M microscope (Zeiss, Germany). CD164 and PDPN were co-stained on the same section and CD146 stained on a separate section to avoid fluorescence overlap.

#### Flow cytometry

The transitional 3D cultures were subjected to flow cytometry analysis for the identification of skeletal associated progenitor markers. Cellular 10% collagen gels were fabricated and cultured for 7 days under hypoxic conditions. Cells were collagenase released (500U) and subjected to flow cytometry analysis. Additionally, 10% collagen gels were embedded within 0.2% collagen gels to form the 3D transitional culture, as previously described. These samples were cultured for a further 7 and 14 days. The 0.2 and 10% collagen gels were separated at each time point and digested in 200U and 500U collagenase I (Sigma), respectively, for 1 h at 37°C. Samples were resuspended in TrypLE (ThermoFisher) for 5 min and washed in PBS. The cells were spun at 1,700 rpm for 5 min and the pellets were resuspended in fresh PBS. Cell viability was assessed using a LIVE/DEAD fixable violet dead stain kit (ThermoFisher) for 30 min at room temperature and fixed in 1% paraformaldehyde for 15 min. Cells were stained with PDPN monoclonal (Thermo Fisher Scientific Cat# 12-9381-41, RRID:AB_1582263, Loughborough, Leicestershire, UK), CD73 monoclonal (Thermo Fisher Scientific Cat# 11-0739-42, RRID:AB_10596508) and CD146 monoclonal antibodies (Thermo Fisher Scientific Cat# 11-1469-42, RRID:AB_2043805) for 1 h at room temperature. Flow cytometry was conducted on a BD LSRFortessa™ (San Jose, CA, USA). Data was analysed using FlowJo™ software (FlowJo, LLC., RRID:SCR_008520, OR, USA).

### Quantification and statistical analysis

Data are expressed as mean ± SEM. Statistical significance was determined using one-way analysis of variance with Fisher’s least significant difference, post hoc corrections applied or Student’s *t* test. Statistical significance is indicated on all graphs as follows: ∗p *<* 0.05, ∗∗p *<* 0.01, ∗∗∗p *<* 0.001 (n = 3; represents number of experimental replicates for each condition). All the statistical details of experiments can be found in the figure legends. All statistical analysis was performed using GraphPad Prism version 6.0f for windows (GraphPad Prism Software, La Jolla, CA, USA, http://www.graphpad.com).

## Data Availability

• All data reported in this paper will be shared by the lead contact upon request. • This paper does not report original code. • Any additional information required to reanalyse the data reported in this paper is available from the lead contact upon request.
