# Supplementary material for: Reprogramming bone progenitor identity and potency through control of collagen density and oxygen tension
Source: iScience. 2022 Mar 11;25(4):104059. doi: 10.1016/j.isci.2022.104059 (PMC8957015; doi:10.1016/j.isci.2022.104059)
Supplement: Document S1. Figures S1, S2, and Table S1 [file mmc1.pdf]

**iScience, Volume *25***

## Supplemental information

# Reprogramming bone progenitor identity and potency through control of collagen density and oxygen tension

**Rawiya Al Hosni, Laurent Bozec, Scott J. Roberts, and Umber Cheema**

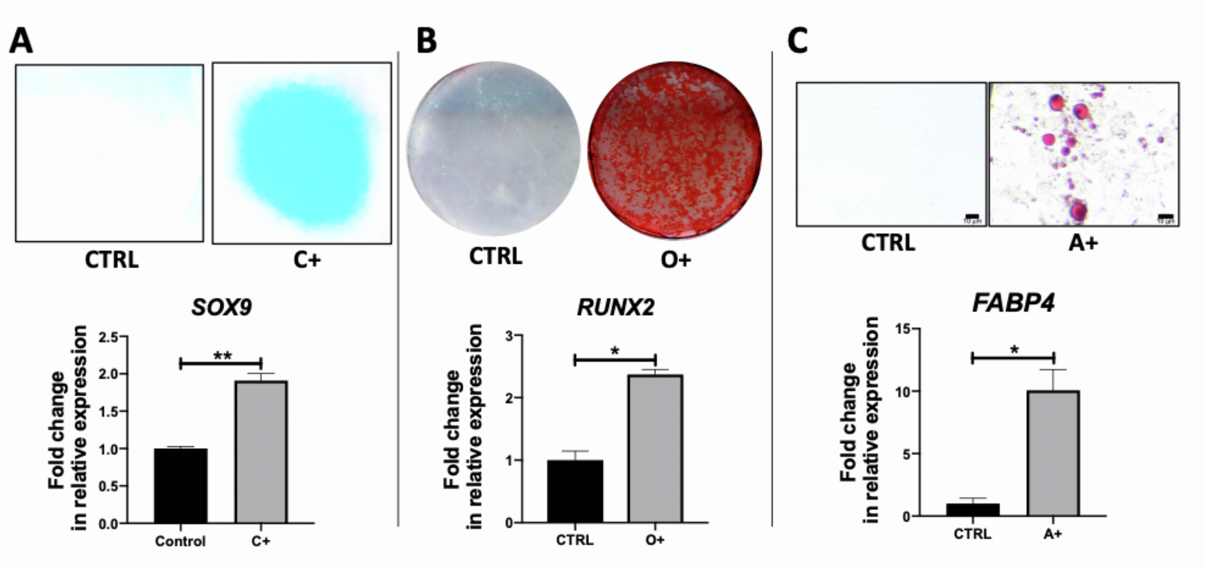


**Figure S1 Characterisation of AMSC potency, Related to Figure 1**. AMSCs were assessed for their ability to differentiate down the chondrogenic, osteogenic and adipogenic lineages. **A)** Chondrogenic differentiation assessed for GAGs detected using Alcian Blue after 7 days and *SOX9* gene expression analysed by qPCR. **B)** An osteogenic differentiation assay was conducted for 21 days, illustrating positive calcium phosphate staining using Alizarin Red with *RUNX*2 expression analysed by qPCR. **C)** An adipogenic differentiation assay was conducted over 21 days, with the presence of fat droplets analysed using Oil Red O stain and *FABP4* expression relative to control analysed using qPCR (scale bar: 10 μm). (Data are presented as the mean ±S.E.M, Statistical analysis performed using Mann-Whitney test; ***P*<0.01; **P*<0.05; n=3).

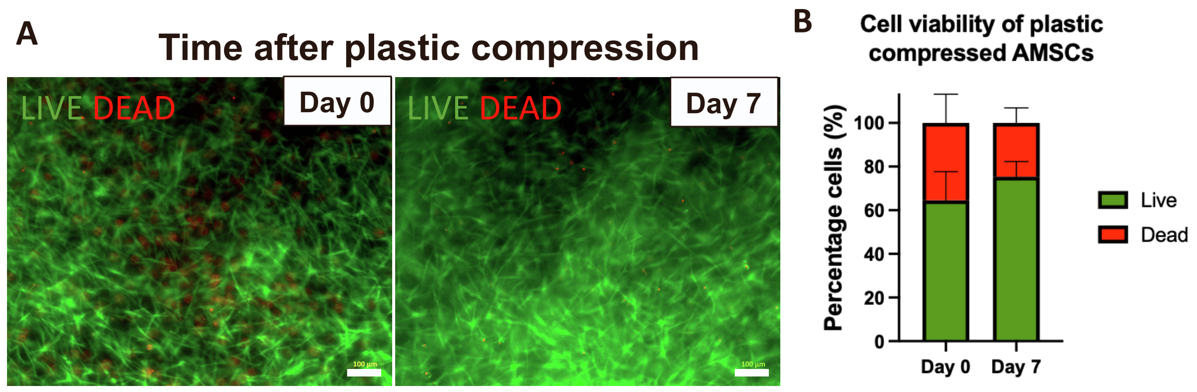


**Figure S2 Cell viability of plastic compressed AMSCs, related to Figure 1**. AMSCs were seeded in 0.2% collagen type I gels and subjected to plastic compression using RAFT™ absorbers to form a 10% collagen type I gel. A LIVE/DEAD assay was performed to assess cell viability. **A)** Representative images of cells stained with a LIVE/DEAD dye at day 0 and day 7 (Scale bar- 100 μM). **B)** Quantification of percent live and dead cells at day 0 and day 7. An increase in cell viability was observed 7 days after plastic compression.

**Table S1** Primer sequences used to conduct gene expression analysis using qPCR, related to STAR Methods.

| **Gene Name** | **Forward (5'-3')** | **Reverse (3'-5')** |
|---|---|---|
| *CD146* | GGAAGGTGTGGGTGAAAGAG | GGACATTCAGGGTGCTCAG |
| *CD164* | CCTTAGCTTTCTCCCGAACG | TGCTGGGTCGTGTTCTTG |
| *CD73* | ACTGGGACATTCGGGTTTTG | CTCTTTGGAAGGTGGATTGC |
| *FABP4* | CATACTGGGCCAGGAATTTG | GGACACCCCCTCTAAGGTT |
| *GAPDH* | GCTCTCTGCTCCTCCTGTTC | CGACCAAATCCGTTGACTCC |
| *NES* | GGCCACGTACAGGACCCTC | CCTCTGGGGTCCTAGGGAAT |
| *PDPN* | CTCTGCTCTTCGTTTTGGGA | GAGTCACCACATCATCTTCGG |
| *PRX1* | CGAGAGTGCAGGTGTGGTTT | GAGCAGGACGAGGTACGAT |
| *RUNX2* | CGCATTCCTCATCCCAGTAT | GCCTGGGGTCTGTAATCTGA |
| *SOX9* | TGGAGACTTCTGAACGAGAGC | CGTTCTTCACCGACTTCCTC |
| *TAZ* | GCTACACTCCCACTTCTTCAG | CGCCATCTCCTTTCTCTCTTC |
| *YAP* | CCCTCGTTTTGCCATGAACC | TGTTGCTGCTGGTTGGAGTT |
